# Association between exclusive breastfeeding and postpartum post-traumatic stress disorder

**DOI:** 10.1186/s13006-022-00519-z

**Published:** 2022-11-23

**Authors:** Jingfen Chen, Xiaolu Lai, Lepeng Zhou, Ravi Retnakaran, Shi Wu Wen, Daniel Krewski, Liping Huang, Meng Li, Ri-hua Xie

**Affiliations:** 1 School of Nursing, Southern Medical University, Guangzhou, China; 2 Leadership Sinai Centre for Diabetes, Mount Sinai Hospital, Toronto, Ontario Canada; 3 Lunenfeld-Tanenbaum Research Institute, Mount Sinai Hospital, Toronto, Ontario Canada; 4 Division of Endocrinology, University of Toronto, Toronto, Ontario Canada; 5 Clinical Epidemiology Program, Ottawa Hospital Research Institute, Ottawa, Ontario Canada; 6 Department of Obstetrics and Gynecology, University of Ottawa, Ottawa, Ontario Canada; 7 School of Epidemiology and Public Health, University of Ottawa, Ottawa, Ontario Canada; 8 McLaughlin Centre for Population Health Risk Assessment, University of Ottawa, Ottawa, Ontario Canada; 9 Risk Science International, Ottawa, Ontario Canada; 10 Department of Obstetrics and Gynecology, Southern Medical University Nanfang Hospital, Guangzhou, China; 11 The Second School of Clinical Medicine, Southern Medical University, Guangzhou, China; 12 Department of Obstetrics, The Seventh Affiliated Hospital, Southern Medical University, Foshan, China; 13 Affiliated Foshan Maternity & Child Healthcare Hospital, Southern Medical University, Foshan, China; 14 School of Nursing, Southern Medical University, Guangzhou, China; 15 The Telfer School of Management, University of Ottawa, Ottawa, Ontario Canada

**Keywords:** Exclusive breastfeeding, Post-traumatic stress disorder, Postpartum mothers, Epidemiologic study

## Abstract

**Background:**

Research on the association between breastfeeding and postpartum post-traumatic stress disorder (PTSD) is sparse. This study aimed to examine the association between exclusive breastfeeding up to 42 days after childbirth and postpartum PTSD.

**Methods:**

An epidemiologic study was conducted in a tertiary hospital in China between October 2019 and October 2020. Eligible mothers were recruited at 3 days after childbirth and assessed using the Post-Traumatic Stress Disorder Checklist – Civilian version (PCL-C) for PTSD at 42 days postpartum. The independent association between exclusive breastfeeding up to 42 days after childbirth and postpartum PTSD was estimated using log-binomial regression models, after adjusting for potential confounders.

**Results:**

Ninety-two of 759 (12.1%) mothers developed postpartum PTSD within 42 days after childbirth. Compared with partially breastfeeding mothers, exclusively breastfeeding mothers had lower risks of postpartum PTSD (relative risk [RR] 0.28; 95% confidence interval [CI] 0.13, 0.59), re-experience (RR 0.48; 95% CI 0.30, 0.76), avoidance (RR 0.55; 95% CI 0.32, 0.97), and hyperarousal (RR 0.52; 95% CI 0.34, 0.78). After adjustment for family support, parity, mode of delivery, perceived birth trauma, early contact / suckling, and rooming-in, associations between exclusive breastfeeding and postpartum PTSD remained significant: the overall PTSD adjusted relative risk [aRR] was 0.31; (95% CI 0.15, 0.66), with a re-experience aRR of 0.48; (95% CI 0.30, 0.77) and hyperarousal aRR of 0.56; (95% CI 0.37, 0.85).

**Conclusion:**

Exclusive breastfeeding up to 42 days after childbirth was associated with reduced risk of postpartum PTSD. While the potential for reverse causation cannot be ruled out, strategies to improve rates of exclusive breastfeeding through teaching, counselling, and support may benefit mothers and their infants by reducing the risk of postpartum PTSD.

## Background

Post-traumatic stress disorder (PTSD), one of the most common psychiatric disorders following a severe traumatic event, is characterized by re-experience, avoidance, and hyperarousal [1]. Childbirth is a complex event that may induce a traumatic reaction, and possibly lead to postpartum PTSD [2]. A meta-analysis of 78 studies found that the prevalence of postpartum PTSD was 3.1% in community samples and 15.7% in at-risk samples [3]. Mothers with postpartum PTSD may develop nightmares about childbirth and be reluctant to engage with their newborns, interfering with breastfeeding [3, 4].

It has been reported that breastfeeding mothers had lower risks of mood disorders such as depression and anxiety than formula-feeding mothers [5–8], especially for mothers who breastfed exclusively and for longer durations [8, 9]. A prospective study that followed 209 women at three time points (during pregnancy, during the first home visit after hospitalization for childbirth and at 3 months postpartum) has revealed that the rate of postpartum depression in women who breastfed (45.9%) within 3 months after childbirth was significantly lower (*p* = 0.029) than for those women who did not do so (54.1%) [6]. On the other hand, previous studies have shown that postpartum depression and PTSD led to lower rates of breastfeeding [4, 10, 11]. In a large-scale study (*n* = 1480), data from 8 weeks to 2 years postpartum indicated that, postpartum PTSD was significantly related to discontinuance of breastfeeding (adjusted odds ratio [aOR] 5.98; 95% confidence interval [CI] 1.79, 19.97) [11]. However, evidence regarding the association between exclusive breastfeeding and postpartum PTSD is sparse. Thus, this study aimed to test the hypothesis that exclusive breastfeeding up to 42 days after childbirth was associated with a lower risk of postpartum PTSD in mothers.

## Methods

### Design and study population

This epidemiologic study was performed in a tertiary hospital in Guangdong, China from October 2019 to October 2020. Mothers were recruited if they were 18 years of age or older with a single full-term live birth and scored less than 13 on the Edinburgh Postnatal Depression Scale (EPDS) [12] at 3 days after childbirth. Mothers were excluded if they met one or more of the following criteria: (1) previous history of a severe physical or psychological trauma; (2) severe obstetric complications (e.g., placenta previa and amniotic fluid embolism); and / or (3) adverse neonatal outcomes (e.g., admission to neonatal intensive care unit and neonatal asphyxia). Written informed consent was obtained from all eligible mothers. Approval from the Research Ethics Committee of the study hospital was obtained (2019-09-01) before commencement of the study.

### Measurement of postpartum PTSD

Mothers were assessed for PTSD at 42 days after childbirth using the 17-item Post-Traumatic Stress Disorder Checklist – Civilian version (PCL-C) [13]. This standardized self-report rating scale measures the key symptoms of PTSD including re-experience, avoidance, and hyperarousal. Mothers were asked to indicate the degree of each symptom related to the current delivery experiences within the last month, ranging from 1 (not at all) to 5 (extremely). A total symptom severity score ranging from 17 to 85 was derived by summing the scores from each of the 17 items (including five items of re-experience, seven items of avoidance, and five items of hyperarousal), with higher scores indicating more severe PTSD symptoms. A score of more than three on each item was regarded as a positive critical value. A threshold of 38 or higher was selected as the optimal cut-point for probable PTSD [14]. Mothers were considered to demonstrate symptoms of re-experience if at least one of the five items in the re-experiencing symptom cluster was positive, and symptoms of avoidance and hyperarousal if at least three avoidance items and two hyperarousal items were positive. The Chinese version of PCL-C has shown good diagnostic agreement, sound reliability (Cronbach’s α above 0.77) and validity [15–17]. Considering that the PCL-C assessed PTSD symptoms in the past month [13], and rate of PTSD was likely the highest at 4 to 6 weeks postpartum [18, 19], we decided to follow up the participants at 42 days postpartum, which also corresponds with the time for mothers to be reviewed routinely in the hospital in accordance with the maternal and child healthcare policy in China.

### Collection of demographic, clinical, and breastfeeding data

A self-administered questionnaire was used to collect demographic and clinical data on the study participants in the obstetric ward at 3 days after childbirth. Demographic data included maternal age, education, occupation, marital status, annual household income, and family support. Clinical data included history of abortion, parity, mode of delivery, perceived birth trauma, sex of neonate, early contact / suckling, as well as rooming-in. Mothers were asked whether they perceived the present childbirth as a traumatic event, with responses of “yes” or “no”. The assessment of early contact / suckling was based on the electronic medical records, which defined early contact / suckling practice as the naked newborn was placed skin-to-skin on their mother’s bare chest and suckled the breast within the first hour after childbirth.

Breastfeeding data including exclusive breastfeeding, partial breastfeeding and exclusive formula feeding were collected at 42 days postpartum. Mothers were asked to recall how they fed their infant from birth to 42 days postpartum, with the options of “only breast milk”, “breast milk and sugar water”, “breast milk and formula”, “only formula”, or “others”. If mothers selected “only breast milk”, they would be further asked: “Has the baby only been fed with mother’s milk since birth? And no other liquid (including water) or solids other than oral rehydration solution, vitamins, minerals and medicines were given to the baby?”. These questions on feed types were asked according to the definition of exclusive breastfeeding given by the World Health Organization [20]. However, the World Health Organization definition refers to exclusive breastfeeding on the previous day, we applied this definition from birth to 42 days postpartum in the present study. If mothers answered “yes”, their babies would be considered to have experienced exclusive breastfeeding up to 42 days after childbirth.

### Data analysis

All analyses were performed using the Statistical Package for the Social Sciences (SPSS 26.0) [21]. Demographic and clinical characteristics of mothers with exclusive breastfeeding and partial breastfeeding were compared using descriptive statistics. A Chi-square test or Mann-Whitney *U* tests was used to compare frequency distributions for categorical variables, and a 2-tailed *t*-test was used to compare continuous variables, if appropriate, with *p* < 0.05 considered statistically significant.

Multiple log-binomial regression analyses were performed to assess the independent association of exclusive breastfeeding up to 42 days after birth on postpartum PTSD, using partially breastfeeding mothers as the reference, and relative risk (RR) and associated 95% CI as the effect measures. Confounders included in the multiple regression models were family support (low / moderate / high), parity (primiparous / multiparous), mode of delivery (vaginal / cesarean delivery), perceived birth trauma (no / yes), early contact / suckling (no / yes), and rooming-in (no / yes).

## Results

### Characteristics of study participants

**Fig. 1:**
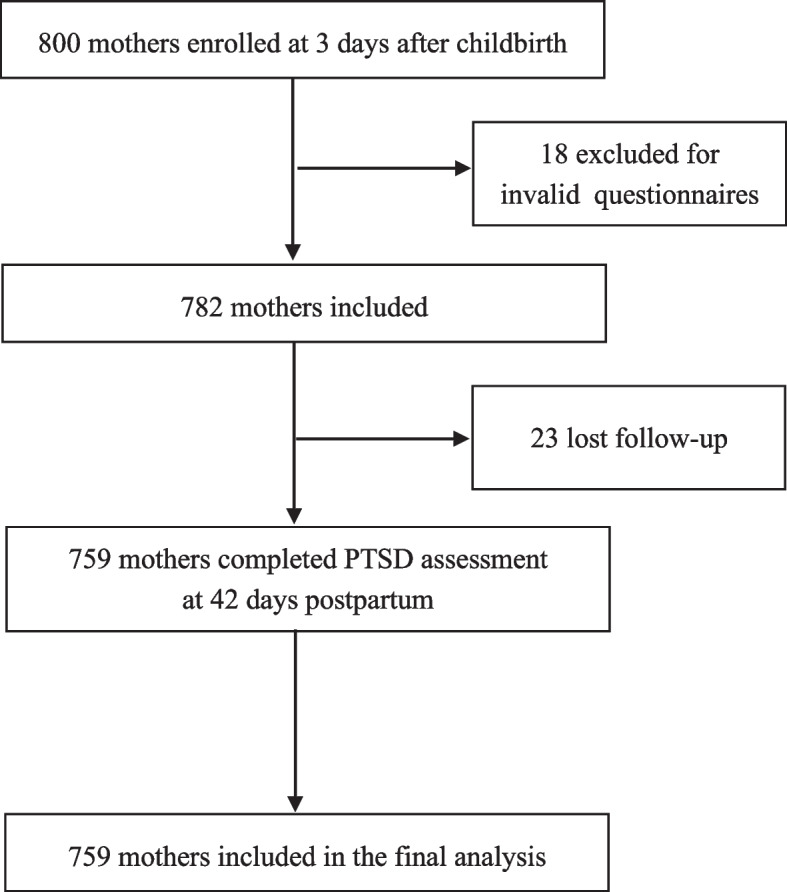
Flowchart showing recruitment of mothers to this study

A total of 800 mothers met our eligibility criteria and were enrolled into this study at 3 days after childbirth. Of them, 18 mothers were excluded because of invalid questionnaires and 23 mothers were lost to follow-up, leaving 759 eligible mothers (94.9%) for the final analysis, including 174 exclusively breastfeeding mothers and 585 partially breastfeeding mothers (see Fig. 1). The demographic and clinical characteristics of the mothers in this study are summarized in Table 1. Maternal age ranged from 20 to 40 years (with a mean age of 28.2 ± 4.8 years). Most mothers had a high school education, were housewives, and had an annual household income of 30,000–100,000 renminbi (RMB). More than half were multiparous and delivered vaginally. Exclusively breastfeeding mothers were less likely to perceive they experienced birth trauma and more likely to have had early contact / suckling, and rooming-in with their babies after childbirth.

**Table 1:** Demographic and clinical characteristics collected at 3 days postpartum by breastfeeding status assessed at 42 days postpartum, Guangdong, China, 2019-2020^a^

Characteristic	Exclusively breastfeeding mothers (*n* = 174)	Partially breastfeeding mothers (*n* = 585)	*P* value^b^
Maternal age (years), mean ± SD	28.4 ± 4.6	28.1 ± 4.8	0.542
Education			0.946
Primary school or below	3 (1.7)	14 (2.4)	
High school	128 (73.6)	425 (72.7)	
College or above	43 (24.7)	146 (25.0)	
Occupation			0.096
Professional	40 (23.0)	118 (20.2)	
Worker and self-employee	48 (27.6)	132 (22.6)	
Housewife	86 (49.4)	335 (57.3)	
Marital status			0.057
Married	163 (92.7)	519 (88.7)	
Unmarried	11 (6.3)	66 (11.3)	
Annual household income (RMB)			0.001
< 30,000	66 (37.9)	149 (25.5)	
30,000-100,000	106 (60.9)	421 (72.0)	
> 100,000	2 (1.2)	15 (2.6)	
Family support			0.017
Low	11 (6.3)	50 (8.6)	
Moderate	85 (48.9)	199 (34.0)	
High	78 (44.8)	336 (57.4)	
History of abortion			0.651
Yes	79 (45.4)	277 (47.4)	
No	95 (54.6)	308 (52.7)	
Parity			0.112
Primiparous	114 (65.5)	241 (41.2)	
Multiparous	60 (34.5)	344 (58.8)	
Mode of delivery			0.076
Vaginal delivery	111 (63.8)	329 (56.2)	
Cesarean delivery	63 (36.2)	256 (43.8)	
Perceived birth trauma			0.034
Yes	31 (17.8)	150 (25.6)	
No	143 (82.2)	435 (74.4)	
Sex of neonate			0.864
Male	92 (42.9)	305 (52.1)	
Female	82 (47.1)	280 (47.9)	
Early contact/suckling			<0.001
Yes	169 (97.1)	437 (74.7)	
No	5 (2.9)	148 (25.3)	
Rooming-in			<0.001
Yes	169 (97.1)	437 (74.7)	
No	5 (2.9)	148 (25.3)	

*Abbreviations*: *SD* standard deviation

^a^ Data are reported as n (%) unless otherwise indicated

^b^ 2-tailed *t* test, Chi-square test, or Mann-Whitney *U* test

### The rate of postpartum PTSD and PCL-C scores in study participants

Ninety-two out of 759 (12.1%) mothers developed postpartum PTSD within 42 days after childbirth. The rate of PTSD was 4.0% in exclusively breastfeeding mothers, much lower than that in partially breastfeeding mothers (14.5%). The total symptom score, scores for re-experience, avoidance, and hyperarousal were all lower in exclusively breastfeeding mothers when compared to partially breastfeeding mothers (Table 2).

**Table 2:** Post-Traumatic Stress Disorder Checklist-Civilian version (PCL-C) total score and sub-clusters scores by breastfeeding status, assessed at 42 days postpartum, Guangdong, China, 2019–2020

Variable	Median (IQR)		*P* value^a^
	Exclusively breastfeeding mothers (*n* = 174)	Partially breastfeeding mothers (*n* = 585)	
PCL-C total symptom score	21 (18, 26)	23 (20, 29)	<0.001
PCL-C symptom sub-clusters scores			
Re-experience	6 (5, 7)	6 (5, 8)	0.005
Avoidance	8 (7, 10)	9 (7, 10)	<0.001
Hyperarousal	7 (5, 9)	8 (6, 11)	<0.001

*Abbreviations*: *PCL-C* Post-Traumatic Stress Disorder Checklist-Civilian version, *IQR* inter quartile range

^a^ Mann-Whitney *U* test

### Results of multiple log-binomial regression analysis

After controlling for potential confounders, the risk of postpartum PTSD in the exclusively breastfeeding mothers remained substantially lower than that in partially breastfeeding mothers, as measured either by the total score or by sub-clusters scores on the PCL-C scale (Table 3).

**Table 3:** Association between exclusive breastfeeding and post-traumatic stress disorder (PTSD), assessed at 42 days postpartum, Guangdong, China, 2019–2020

Variable	No. (%)		RR (95% CI)	
	Exclusively breastfeeding mothers (*n* = 174)	Partially breastfeeding mothers (*n* = 585)	Crude	Adjusted^a^
PTSD	7 (4.0)	85 (14.5)	0.28 (0.13, 0.59)	0.31 (0.15, 0.66)
Re-experience	20 (11.5)	131 (22.4)	0.48 (0.30, 0.76)	0.48 (0.30, 0.77)
Avoidance	13 (7.5)	79 (13.5)	0.55 (0.32, 0.97)	0.65 (0.37, 1.15)
Hyperarousal	24 (13.8)	145 (24.8)	0.52 (0.34, 0.78)	0.56 (0.37, 0.85)

*Abbreviations*: *No*. number, *RR* relative risk, *CI* confidence interval, *PTSD* post-traumatic stress disorder

^a^ Adjusted for family support, parity, mode of delivery, perceived birth trauma, early contact/suckling, and rooming-in

## Discussion

### Main findings

In this epidemiologic study, we found that the risk of postpartum PTSD among exclusively breastfeeding mothers was substantially lower than among partially breastfeeding mothers at 42 days after childbirth, as reflected in either the total PCL-C score or the sub-clusters scores for re-experience, avoidance, and hyperarousal. This association remained after excluding women with a high EPDS score (greater than or equal to 13) at 3 days after childbirth and adjusting for several potential confounding factors, including family support, parity, mode of delivery, perceived birth trauma, and early contact / suckling as well as rooming-in.

### Interpretations

The overall rate of postpartum PTSD in our study was 12.1%, which is within the range of 1–30% shown in a meta-analysis that included 15,637 women [3]. However, a survey involving 1136 Chinese mothers showed that the rate of PTSD was 6.1% at 6 to 8 weeks after childbirth, as assessed by the Perinatal Post-traumatic Stress Questionnaire [22]. This difference might be due to the different measurement instruments, as another Chinese study by Wang et al. [23] found a similar rate (11.6%) of postpartum PTSD evaluated by the PCL-C as is reported here. On the other hand, the discrepancy in the rate of PTSD could be attributed to differences in the duration of the follow-up periods. A longitudinal study that measured the rates of perinatal PTSD at three time points (pregnancy, 4 to 6 weeks postpartum, and 6 months postpartum) indicated that PTSD peaked at 4 to 6 weeks postpartum (11.9%) and subsequently declined at 6 months postpartum (9.2%) [18]. Thus, the higher PTSD rate in our study compared to the other Chinese study by Liu et al. [22] may be due to the different evaluation instruments and assessment times.

In our study, all mothers breastfed up to 42 days postpartum, benefiting from the promotion of breastfeeding in the study hospital in recent years. For instance, prenatal and postnatal breastfeeding education programs were provided to the perinatal women and their families by the obstetric team who were trained in the core curriculum for lactation consultant practice [24]. This study hospital has also been equipped with breastfeeding rooms under the launch of the “10 m^2^ of Love” campaign in accordance with local health administrative requirements [25]. However, the exclusive breastfeeding rate was still low in the present study. The results of our study sample are consistent with a 2018 national cross-sectional survey (*n* = 5237) in China, in which the partial breastfeeding and exclusive breastfeeding rates were 96.3% and 33.6%, respectively, within the first month postpartum [26].

Although no study to date has specifically assessed the association between exclusive breastfeeding and postpartum PTSD, previous studies suggested that breastfeeding might reduce the risk of mental health problems in postpartum women [5–8, 10]. In the present study, exclusive breastfeeding within 42 days after childbirth was associated with a reduced risk of postpartum PTSD, which may be mediated through the regulation of stress biomarkers such as cortisol [27–29]. One study has shown that women with PTSD had very high levels of cortisol in the postpartum period [30], raising the possibility that postpartum PTSD might be linked to the dysregulation of cortisol. Another study has found that lower cortisol levels after breastfeeding were associated with exclusive breastfeeding practices [31]. These observations may partially explain the association between exclusive breastfeeding and postpartum PTSD.

The association between exclusive breastfeeding and postpartum PTSD symptoms may also be mediated by oxytocin, which has been observed to provide therapeutic benefits to patients diagnosed with traumatic stress-related diseases, especially PTSD [32]. In response to suckling during breastfeeding, oxytocin released into the systemic circulation may play an important role in improving maternal mental health [33], accompanied by beneficial effects on mothers’ mood, affect, and stress, providing a calm and non-anxious state. Similar effects on emotion and stress as seen for breastfeeding have also been demonstrated in a randomized double-blind controlled trial administering oxytocin intranasally compared to a placebo [34], suggesting that the effectiveness of breastfeeding on maternal mood may be related to an increase in oxytocin levels.

### Strengths and limitations

To the best of our knowledge, this is the first study on the association between exclusive breastfeeding and postpartum PTSD. We excluded mothers with a history of or current psychiatric disorders including PTSD and postpartum depression at recruitment and ascertained only new PTSD cases. We also adjusted for potential confounding factors to minimize the impact of important covariates on the observed association between exclusive breastfeeding and PTSD.

This study also has certain limitations. First, the PCL-C has been frequently applied in the general population, but it has not been widely used in postpartum mothers. We are therefore unsure if it can ascertain all PTSD symptoms in our study participants. Second, study participants were recruited in a single center in China. Replication is needed to determine whether and to what extent findings from this study can be generalized to other populations. Third, since no infants were fed solely with formula in this study, we cannot explore possible associations between PTSD and formula feeding. Lastly, despite adjusting for confounding factors, residual confounding caused by unmeasured or unknown factors may still exist, given our limited knowledge on the mechanisms underlying the association between exclusive breastfeeding and PTSD.

## Conclusion

Our large sample epidemiologic study suggests that exclusive breastfeeding up to 42 days after childbirth may be associated with a reduced risk of postpartum PTSD. While the potential for reverse causation cannot be ruled out, strategies to improve rates of exclusive breastfeeding through teaching, counselling, and support may benefit mothers and their infants by reducing the risk of postpartum PTSD.

## Data Availability

The datasets used and / or analyzed during the current study are available from the corresponding author upon reasonable request.

## Abbreviations

aOR: Adjusted odds ratio
aRR: Adjusted relative risk
CI: Confidence interval
EPDS: Edinburgh Postnatal Depression Scale
IQR: Inter-quartile range
PCL-C: Post-Traumatic Stress Disorder Checklist-Civilian version
PTSD: Post-traumatic stress disorder
RR: Relative risk
SD: Standard deviation
